# Generation of Inducible Immortalized Dendritic Cells with Proper Immune Function *In Vitro* and *In Vivo*

**DOI:** 10.1371/journal.pone.0062621

**Published:** 2013-04-23

**Authors:** Cornelia Richter, Sebastian Thieme, Joanna Bandoła, Magdalena Laugsch, Konstantinos Anastassiadis, Sebastian Brenner

**Affiliations:** 1 Department of Pediatrics, University Clinic Carl Gustav Carus, Technische Universitaet Dresden, Dresden, Germany; 2 Stem Cell Engineering, BIOTEC, Technische Universitaet Dresden, Dresden, Germany; 3 Center for Regenerative Therapies Dresden; Technische Universitaet Dresden, Dresden, Germany; University of Bergen, Norway

## Abstract

Dendritic cells are the professional antigen presenting cells of innate immunity and key players in maintaining the balance of immune responses. Studies with dendritic cells are mainly limited by their low numbers *in vivo* and their difficult maintenance *in vitro*. We differentiated bone marrow cells from transgenic mice expressing an inducible SV40 large T-antigen into dendritic cells. When immortalized by dexamethasone and doxycycline, these cells were stable in long-term culture. In the absence of dexamethasone and doxycycline (de-induction), dendritic cells displayed properties of primary cells, characterized by expression of classical dendritic cell surface markers CD11c, CD11b, MHCII, CD40 and CD86. Furthermore, de-induced lipopolysaccharide activated dendritic cells secreted IL-1β, IL-6, TNFα and IL-12. De-induced, Ovalbumin-loaded dendritic cells polarize CD4^+^ T cells into Th1, Th17 and Th2 cells, indicating their correct antigen presenting property. Consistent with intratracheal application of Ovalbumin-loaded primary dendritic cells into mice, the application of de-induced dendritic cells resulted in recruitment of lymphocytes to the lungs. In summary, we successfully expanded dendritic cells using conditional immortalization. The generated dendritic cells demonstrate the characteristic immunophenotype of primary dendritic cells and will facilitate further studies on immunomodulatory properties of dendritic cells.

## Introduction

As the sentinels between innate and adaptive immunity, dendritic cells maintain the balance of pathogen defense and tolerance to self proteins. In order to fulfill this task, dendritic cells are located in a variety of lymphatic and non-lymphatic tissue where they continuously sense for foreign molecules. Dendritic cells were discovered in the 1970s as immune cells with unique properties distinct from macrophages and monocytes [1]. During the last decades, murine dendritic cells were classified into five main subsets according to their origin, localization and expression profile [2]. Conventional dendritic cells (cDCs) comprise the CD8α-type cDCs [3]–[5] and the CD11b-type cDCs [6], [7], plasmacytoid dendritic cells (pDCs) [8], [9], Langerhans cells in the skin [10] and inflammatory, monocyte-derived dendritic cells (Mo-DCs) [11].

Equipped with a variety of pathogen recognition receptors (PRRs), dendritic cells discriminate between self and non-self molecules [12], [13]. After the recognition of foreign pathogen-associated molecular patterns (PAMPs) via Toll-like receptors (TLRs), dendritic cells switch to defense mode, engulf foreign proteins and present the processed peptides to naïve T cells in the draining lymph nodes. The complete activation of T cells requires three signals provided by dendritic cells. After presentation of foreign peptides via MHC class II, co-stimulatory molecules such as CD80 and CD86 are up-regulated. Upon dendritic cell maturation, pro-inflammatory cytokines are secreted and naïve T cells are polarized into specific CD4^+^ effector T cells [14]–[17]. Depending on dendritic cell secreted cytokines, naïve T cells polarize into Th1, Th2 or Th17 effector T cells. An acute inflammatory response, characterized by Th1 effector cells, is mainly mediated by dendritic cell derived cytokine IL-12p70. Allergen mediated immune response is characterized by induction of Th2 cells, polarized by IL-10 producing dendritic cells or IFNα producing pDCs [18]. In contrast to acute inflammatory response, IL-23 induced Th17 effector cells are more related to chronic inflammation and are connected to tumorigenesis [19], [20].

In addition to the polarization of CD4^+^ effector T cells, dendritic cells with expression of CD8α have the capability to induce cytotoxic T cell response by a process known as cross-presentation [21]. Although dendritic cells are not directly infected with the pathogen, they can present exogenous antigens via MHC class I molecules and consequently activate naïve CD8^+^ T cells to polarize into cytotoxic T cells. Cross-presentation and activation of cytotoxic T cells is important for the defense against intracellular pathogens and tumor cells.

During the last years, the potential and the important role of dendritic cells in orchestrating the adaptive immune response were examined in more detail. Elaborate investigations of dendritic cells for immunotherapies need standardized, reproducible experimental conditions. In the following study, we generated conditionally immortalized dendritic cells with classical dendritic cell properties and a stable phenotype long term. This dendritic cell line will enable functional studies to elucidate immune regulation.

## Results

### Generation of induced-immortalized dendritic cells with an immature phenotype

**Figure 1:**
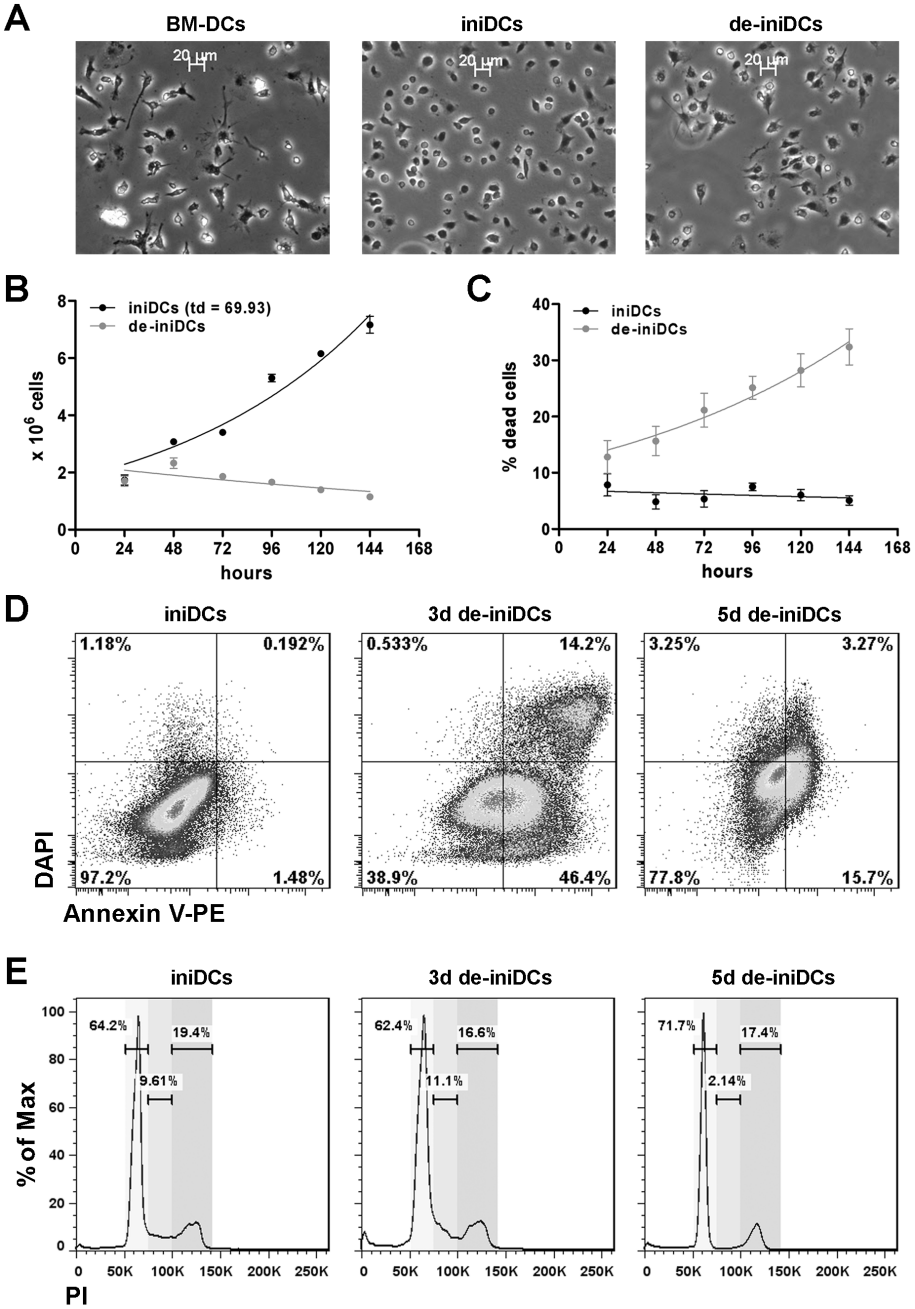
Morphology, cell cycle and proliferation. (A) Microscopic images of BM-DCs, iniDCs and de-iniDCs 3-days after de-induction at 10× magnification. Both, BM-DCs and de-iniDCs show an adherent phenotype with the typical formation of dendrites. (B) Proliferation of iniDCs and de-iniDCs was analyzed by counting the cells in a haemocytometer over a time period of 6 days. (C) Percentage of dead cells counted over a time period of 6 days. (D) Apoptosis and necrosis of iniDCs, 3- and 5-days cultured de-iniDCs were analyzed using anti-AnnexinV-PE antibody and DAPI. Dot blots display AnnexinV and DAPI stained cells. (E) For cell cycle analysis, iniDCs, 3- and 5-days cultured de-iniDCs were stained with PI and analyzed by flow cytometry. Cell cycle stages G1 (left peak), S (middle) and G2 (right peak) were calculated with the Dean-Jett-Fox model using FlowJo software. Proliferation, apoptosis and cell cycle analyses were performed in three independent experiments. For apoptosis and cell cycle analysis the result of a representative experiment is given.

Primary dendritic cells are difficult to culture for a long period of time without loosing their immature state and their characteristic properties. To overcome this problem, we isolated bone marrow cells from an immorto-mouse with a tetracycline regulated expression of SV40 large T-antigen and differentiated those cells into dendritic cells with GM-CSF for 7 days. The cells were then treated with dexamethasone (Dex) and doxycycline (Dox) to induce immortalization. We termed the cells induced-immortalized dendritic cells (iniDCs). After 2 weeks in culture, obvious differences in cell morphology were visible. Dex/Dox treatment resulted in small, round-shaped, less adherent cells (iniDCs) in comparison to non-treated bone marrow derived dendritic cells (BM-DCs; Figure 1A). Next, iniDCs were de-induced (in the absence of Dex/Dox) for 3–5 days to test whether they restore the morphologic phenotype of BM-DCs. And indeed, we observed that de-induced cells (de-iniDCs) display similar morphology as BM-DCs (Figure 1A).

To gain information about the proliferation properties of the iniDCs and de-iniDCs, we counted viable and dead cells. We observed a doubling time of about 70 hours for iniDC (Figure 1B). Proliferation of de-iniDCs stopped 2–3 days after cessation of Dex/Dox, followed by a decrease in cell number (Figure 1B) and an increase of dead cells (Figure 1C). Using CFSE staining we confirmed a constant proliferation rate of iniDCs and a stop of proliferation after de-induction of iniDCs (data not shown). In parallel, apoptosis and necrosis in iniDCs and de-iniDCs was analyzed using anti-Annexin V and DAPI staining. As expected, de-induction of immortalization resulted in increased apoptosis (iniDCs 1.48%, 3d de-iniDCs 15.7% and 5d de-iniDCs 46.4%) and necrosis (iniDCs 0.192%, 3d de-iniDCs 3.27% and 5d de-iniDCs 14.2%) during prolonged culture (Figure 1D). Next, we performed cell cycle analysis via Propidium Iodide (PI) staining. The iniDCs and de-iniDCs displayed differences in the cell cycle stages G1, S and G2. Whereas iniDCs and 3-days cultured de-iniDCs showed only minor differences (G1: 64.2% vs. 62.4%, S: 9.61% vs. 11.1% and G2: 19.4% vs. 16.6%), 5-days cultured de-iniDCs show strongly reduced DNA synthesis (stage S: 2.14%) and accumulate in the G1 stage (G1: 71.7%; Figure 1D).

The iniDCs were found to be stable in long term culture (>25 passages) and the function of iniDCs and de-iniDCs was not affected after several freeze/thaw cycles.

#### Dendritic cell subpopulations are distinguished by their specific cell surface marker profile

**Figure 2:**
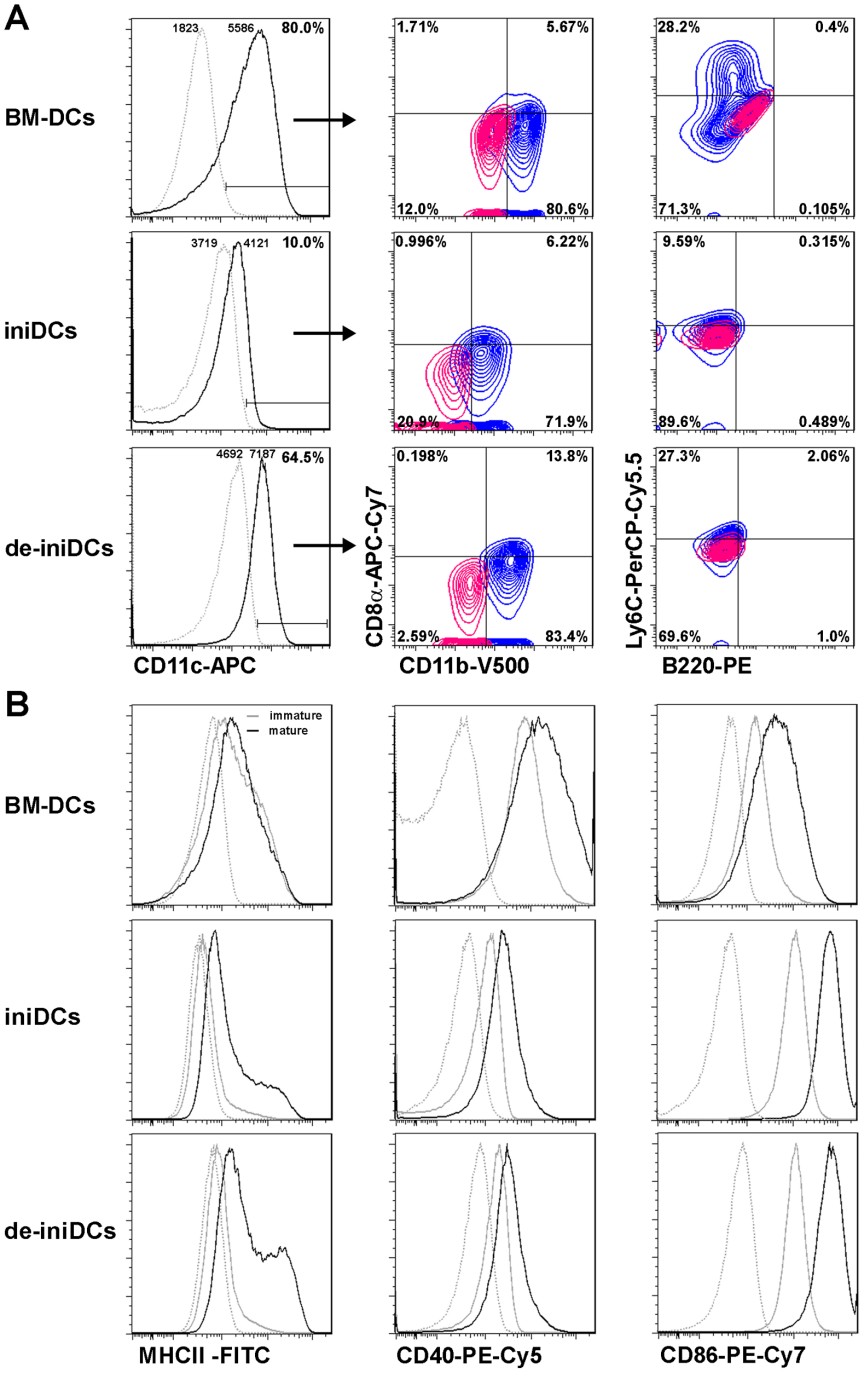
Dendritic cell surface marker expression. (A) BM-DCs, iniDCs and 3-days cultured de-iniDCs were stained with antibodies against the dendritic cell subset markers CD11c, CD8α, CD11b, B220 and Ly6C. CD11c^+^ cells (black curve) were further gated for CD8α and CD11b, Ly6C and B220 (contour blots). Gates for CD8α and CD11b, Ly6C and B220 were set on the respective unstained control (red). (B) Immature and mature BM-DCs, iniDCs and de-iniDCs were stained for MHCII, CD40, and CD86. Dead cells (DAPI staining) and cell doublets were excluded. Histograms show the isotype control (grey, dotted), immature cells (grey) and LPS-matured cells (black). The result of one representative experiment is given.

Due to the fact that the iniDCs were differentiated with GM-CSF from bone marrow cells, we expected a cell surface marker profile for de-iniDCs similar to conventional dendritic cells. Investigating the phenotype of the de-iniDCs, cells were cultured for 5 days in the absence of Dex/Dox. Subsequently, immunostaining with the dendritic cell subset markers CD11c, CD8α, CD11b, B220 and Ly6C was performed for iniDCs, de-iniDCs and BM-DCs. High expression of CD11c and CD11b was detected in de-iniDCs and BM-DCs, whereas CD8α single positive cells were not detectable (Figure 2A). In contrast, we detected in all CD11b^+^ cells a shift of the MFI towards CD8α expression (Figure 2A). Furthermore, a portion of de-iniDCs and BM-DCs are positive for Ly6C, but we detected no plasmacytoid dendritic cells, which are double positive for Ly6C and B220 (Figure 2A). In contrast to de-iniDCs, iniDCs displayed strongly reduced CD11c and Ly6C expression and showed diminished CD11b expression.

The immature phenotype of dendritic cells is characterized by low expression of MHCII and co-stimulatory molecules. To study the phenotype of our dendritic cells, we stained immature and LPS-matured iniDCs and de-iniDCs with fluorochrome-conjugated antibodies against the maturation markers MHCII, CD40 and CD86. While we detected low expression of MHCII in non stimulated iniDCs and de-iniDCs, LPS stimulation results in enhanced expression of this molecule (Figure 2B). Although CD40 and CD86 were already expressed in non stimulated iniDCs and de-iniDCs, both were strongly up-regulated during maturation with LPS. In contrast to dendritic cell markers CD11c and CD11b (Figure 2A) and the MHCII molecule (Figure 2B), expression of CD40 and CD86 was found to be independent of Dex/Dox treatment (Figure 2B).

### Cytokine secretion of de-iniDCs is comparable to that of bone marrow derived dendritic cells

**Figure 3:**
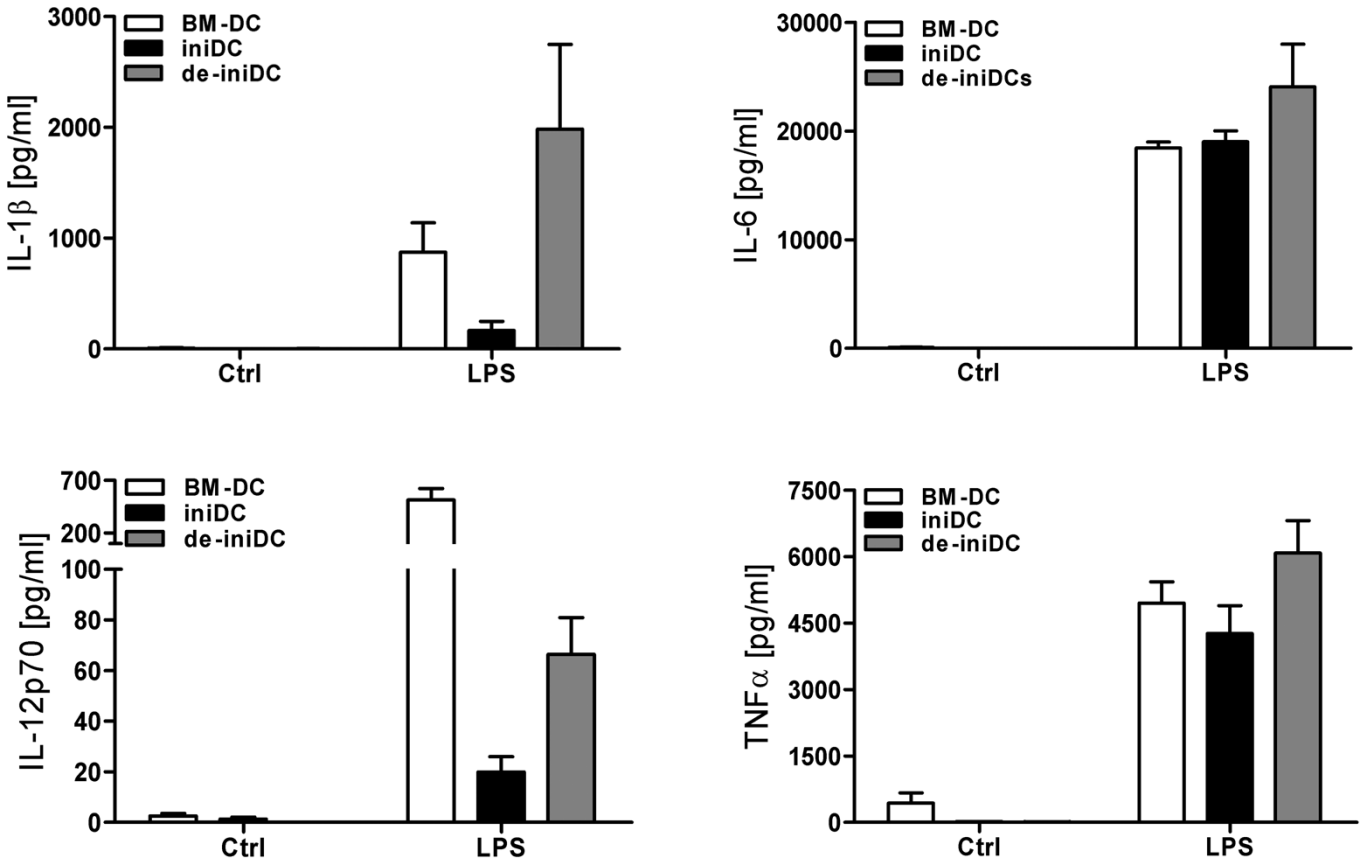
Cytokine profile of dendritic cells. BM-DCs, iniDCs and de-iniDCs were stimulated with LPS (1 µg/mL) for 24 hours. Secretion of IL-1β, IL-6, IL-12p70 and TNFα was measured in the supernatant of non-induced BM-DCs (white bars), iniDCs (black bars) and de-iniDCs (grey bars) via CBA. For each cytokine the mean ± SEM of three independent experiments is given.

Dendritic cells, activated via their Toll-like receptors (TLRs) produce a variety of cytokines such as IL-1β, IL-6, TNFα and IL-12. Therefore, we stimulated iniDCs and BM-DCs with LPS (TLR4) for 24 hours. The secreted cytokines were measured in the cell culture supernatant. We detected high levels of IL-1β, IL-6, IL-12p70 and TNFα in the supernatant of BM-DCs (Figure 3, white bars), whereas iniDCs showed markedly reduced cytokine levels of IL-1β and IL-12p70 (Figure 3, black bars). To test whether our de-iniDCs produced cytokine levels comparable to BM-DCs, we measured the cytokines after stimulation with LPS in 3-days de-induced cells. We found high levels of IL-1β, IL-6 and TNFα, comparable with those of bone marrow derived dendritic cells (Figure 3, grey bars), whereas we detected lower level of IL-12p70 (Figure 3). However, IL-12p70 expression was well detectable by intracellular staining with a specific antibody against the IL-12 subunit p35 (see below). IL-2, IL-4, IL-5, IL-13, IL-17A and IFNγ expression was not detected in the supernatant (data not shown).

### De-iniDCs polarize naïve T cells into CD4^+^ effector T cells and activate CD8^+^ T cells via cross-presentation

**Figure 4:**
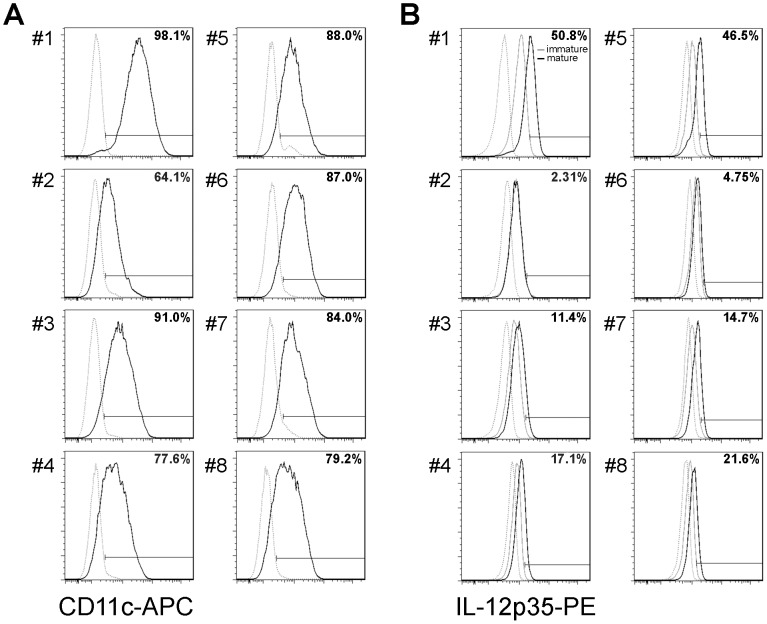
CD11c expression and IL-12 production in single cell clones. De-iniDC single cell clones were stimulated with LPS or left untreated for 24 hours in the presence of the protein transport inhibitor Monensin. Afterwards, cells were stained for the surface marker CD11c, permeabilized and stained for intracellular IL-12. (A) CD11c expression (black) of LPS stimulated cells is displayed. (B) Intracellular IL-12 expression level of CD11c^+^ LPS stimulated (black) and non-stimulated cells (grey) are shown. Isotype control is displayed as grey, dotted curve (A, B).

Our results demonstrate the successful generation of conditionally immortalized dendritic cells. However, the iniDCs are a heterogeneous dendritic cell population with differences in the levels of surface marker expression and cytokine secretion. To obtain a dendritic cell line with defined properties, we generated single cell clones of iniDCs. Stably growing single cell clones were cultured continuously and analyzed for their CD11c expression and IL-12 secretion as key markers for dendritic cell phenotype. Since we detected low levels of IL-12 in the supernatant, we measured this cytokine using intracellular staining. We selected 8 single cell clones, de-induced them for 3 days and stained for CD11c surface expression and intracellular IL-12 level after LPS stimulation. We detected variances in CD11c expression levels (Figure 4A) and IL-12 production (Figure 4B) among the different cell clones. While all cell clones demonstrated a homogeneous shift towards higher CD11c expression as measured by median fluorescence intensity, CD11c positive cells ranged from about 64% to almost 100% compared to isotype control (Figure 4A). IL-12 expression level was found in 2% to 50% of CD11c positive cells, stimulated with LPS (Figure 4B), while the level of CD11c expression did not correlate with the IL-12 level. The maturation of de-iniDC clones during LPS stimulation was confirmed by expression of MHCII, CD86 and CD40 (data not shown). For further characterization of the de-iniDCs, we used the single cell clone #1 exhibiting highest CD11c and IL-12p35 expression (Figure 4A und B, #1).

**Figure 5:**
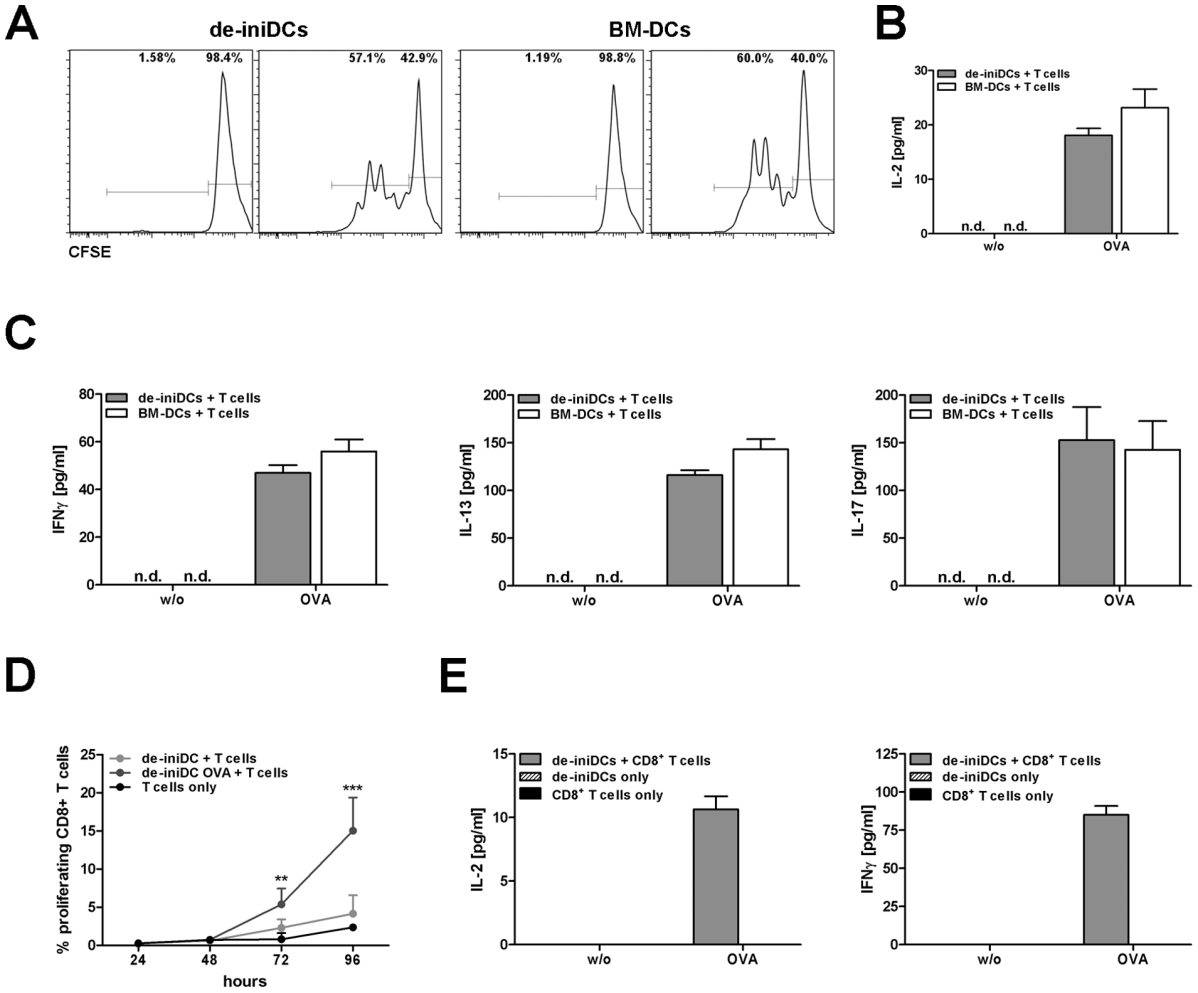
Antigen presentation of de-iniDC clone #1 and BM-DCs to T cells. De-iniDC clone #1 or BM-DCs were incubated with OVA (13.5 µg/mL) for 24 hours prior to co-culture with OTII/CD45.1 CD4^+^ T cells or OTI CD8^+^ T cells. (A) Proliferation of CD4^+^ T cells was measured using CFSE staining and analyzed by flow cytometry. (B) Secretion of IL-2 was measured with CBA. (C) CD4^+^ T cell secreted cytokines IFNγ, IL-13 and IL-17 were measured in the cell culture supernatant using CBA after 48 hours. (D) Proliferation of CD8^+^ T cells was measured using CFSE staining and flow cytometry. (E) CD8^+^ T cell secreted cytokines IL-2 and IFNγ were measured in the supernatant using CBA after 48 hours. Results of three to four independent experiments are given as mean ± SEM, (n.d.) not detectable. Statistical significance is indicated, *(P<0.05), **(P<0.01) and ***(P<0.001).

To test whether our de-iniDC clone #1 is able to present antigens to naïve T cells, we analyzed the proliferation and cytokine secretion (IFNγ, IL-13, IL-17) of CD4^+^ T cells in co-culture experiments. Bone-marrow derived CD11c^+^ DCs were used as positive control. The induction of T cell proliferation by OVA pre-loaded de-iniDC clone #1 as measured by CFSE staining was comparable to the induction by OVA pre-loaded BM-DCs (Figure 5A). Additionally, we detected increased IL-2 secretion as proliferation marker (Figure 5B). Similar to OVA-loaded BM-DCs, our de-iniDC clone polarized CD4^+^ T cells into Th1, Th2 and Th17 types, characterized by the secretion of IFNγ (46.99±3.244 pg/ml), IL-13 (116.1±5.195 pg/ml) and IL-17 (152.7±34.66 pg/ml; Figure 5C). When not in co-culture, T cells, de-iniDC clone #1 or BM-DCs alone do not proliferate and do not secrete IFNγ, IL-17 and IL-13 (data not shown).

Our de-iniDCs also express CD8α (Figure 2A). To proof whether they have cross-presentation capacity, we co-cultured OVA-pulsed de-iniDC clone #1 with CD8^+^ T cells and detected increased T cell proliferation (Figure 5D). In the co-culture supernatant with OVA-loaded de-iniDC clone #1 we found enhanced IL-2 and IFNγ levels (Figure 5E). In summary, our de-iniDCs present antigens to CD4^+^ effector and CD8^+^ cytotoxic lymphocytes.

### Intratracheally applied de-iniDCs induce adaptive immunity in the lung

**Figure 6:**
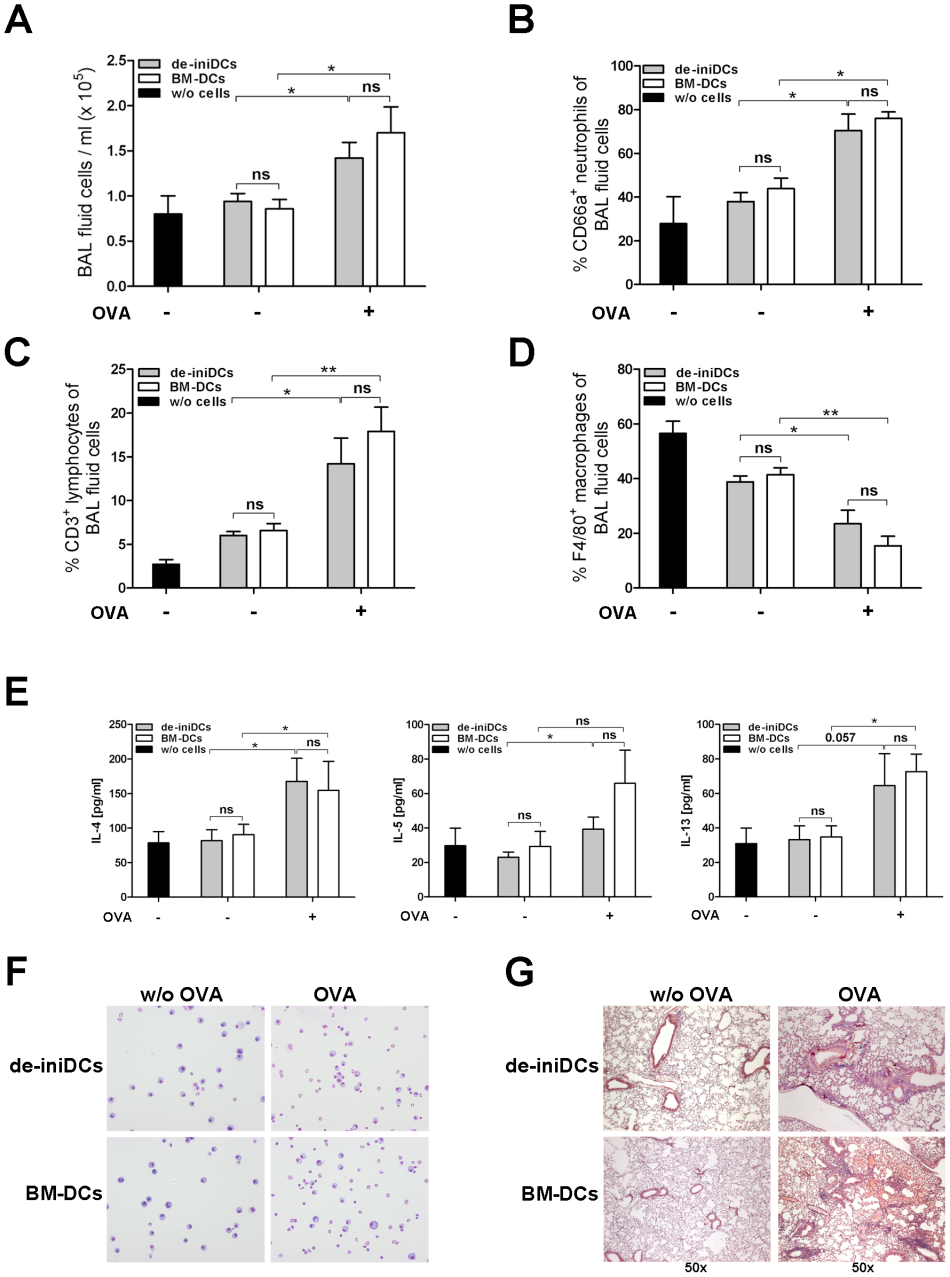
*In vivo* immune response induced by de-iniDCs and BM-DCs. (A) 48 hours after intratracheal application of cells, BAL fluid was collected and cells were counted in a haemocytometer. (B) The percentage of the CD66a^+^ neutrophils in the BAL fluid was analyzed by flow cytometry. (C) Percentage of CD3^+^ T cells in the BAL fluid of provoked mice were analyzed by flow cytometry. (D) Numbers of F4/80^+^ macrophages in the BAL fluid were analyzed by flow cytometry. (E) T cell cytokine secretion was measured in the BAL fluid by CBA. (F) May-Grünwald-Giemsa stained cytospin preparations demonstrate recruited eosinophils. (G) Paraffin-embedded lung sections were stained with Hematoxylin and Eosin. Results are expressed as mean ± SEM from 5 mice per group. Statistical significance is indicated, *(P<0.05) and **(P<0.01).

To investigate, whether de-iniDCs are functional *in vivo*, we applied OVA-loaded or mock (PBS) treated de-iniDC clone and CD11c^+^ BM-DCs to the lungs of OTII/CD45.1 mice by intratracheal application. We detected significantly increased cell numbers in the BAL fluid of mice treated with OVA-loaded de-iniDCs (1.42×10^5^±0.174) and BM-DCs (1.7×10^5^±0.286), respectively compared to mice that received mock-treated cells (de-iniDCs: 0.86×10^5^±0.103; BM-DCs: 0.94×10^5^±0.087; Figure 6A). Control mice, which received PBS without cells showed comparable cell numbers in the BAL fluid as mice that received mock treated cells (Figure 6A, black bar, 0.8×10^5^±0.2). Analysis of BAL fluid cells by flow cytometry revealed significantly higher percentages of CD66a^+^ neutrophils in mice treated with OVA-loaded de-iniDCs (70.42±7.55%) or BM-DCs (75.95±3.02%; Figure 6B) compared to mice that received either mock-treated cells or PBS without cells (de-iniDCs: 37.94±4.04%; BM-DCs: 43.84±4.84%; w/o cells: 27.8±12.4%; Figure 6B).

Furthermore, we used a mouse model for asthma to investigate T cell activation by de-iniDCs *in vivo*. OVA-loaded de-iniDC clone #1 and CD11c^+^ BM-DCs were intracheally applied to C57BL/6 mice. After provocation with OVA aerosol the BAL fluid of mice which received OVA-loaded de-iniDC clone #1 or BM-DCs contained significantly higher numbers of CD3^+^ T cells compared to the BAL fluid of mice that received mock-treated cells (Figure 6C). In contrast, number of macrophages decreased significantly in the BAL fluid of mice receiving OVA-loaded de-iniDC clone #1 or OVA-loaded BM-DCs (Figure 6D). Because the experimental asthma model is Th2 (allergy) prone, we measured corresponding cytokines in the BAL fluid. In mice that received OVA-loaded de-iniDC clone #1 or OVA-loaded BM-DCs, we detected significantly increased levels of IL-4, IL-5 and IL-13 compared to mice that received mock-treated cells (Figure 6E). Additionally, we detected increased numbers of eosinophils in cytospin analyses from mice that received OVA-loaded de-iniDC clone #1 or BM-DCs (Figure 6F). We found massive infiltration of immune cells around the bronchi in H&E stained histology sections of the lungs (Figure 6G). Using this murine asthma model, we showed that de-iniDCs are functional antigen-presenting cells *in vivo*.

### Efficient lentiviral vector mediated transgene expression in iniDCs without immunogenic side effect

**Figure 7:**
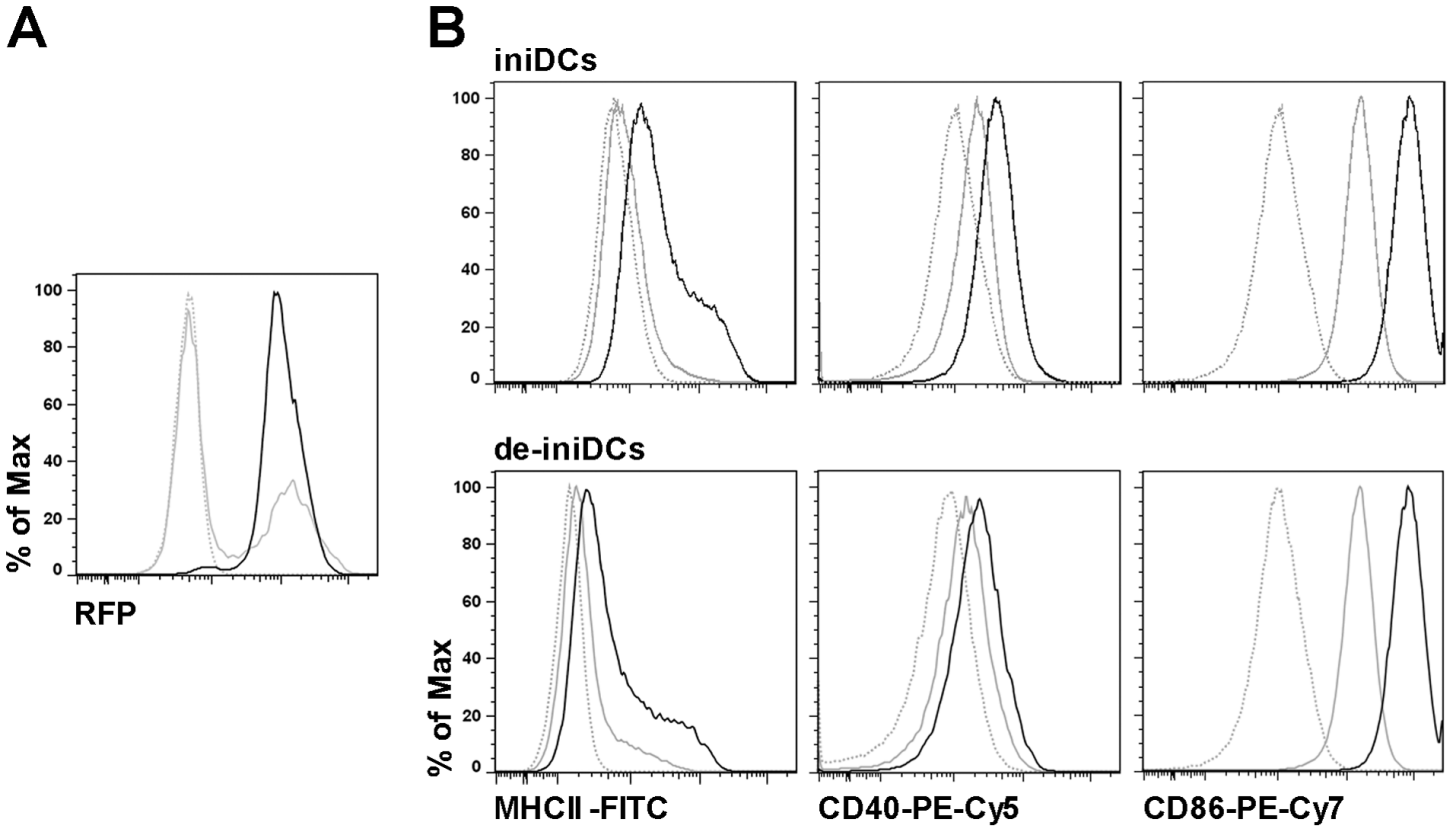
Lentiviral vector mediated transgene expression in iniDCs. (A) RFP expression level was measured in untransduced (grey dotted) and lentiviral vector particle-transduced iniDCs before (grey) and after (black) puromycin selection. (B) Expression level of maturation markers MHCII, CD40 and CD86 were determined in transduced iniDCs and after their deinduction (de-iniDCs) using flow cytometry. Transduced iniDCs and de-iniDCs (grey) and LPS-stimulated transduced iniDCs and de-iniDCs (black) are shown. Isotype controls are displayed as grey dotted lines. One representative experiment out of 3 is shown.

Functional studies of dendritic cells are not only limited by their low frequency or limited survival rate *in vitro*, but also genetically manipulated primary cells may be activated by the introduced DNA/RNA or the transfection procedure itself. To analyze the transduction efficiency of iniDCs and to investigate whether they get activated during this process, we transduced the iniDC clone #8 with lentiviral vector particles enabling RFP expression. We achieved a transduction rate of about 30–40%, quantified by the expression level of RFP via flow cytometry (Figure 7A, grey line). Puromycin selection of transduced cells for 72 hours resulted in ∼98% RFP positive cells (Figure 7A, black line). Transduced iniDCs and de-iniDCs were stained for maturation markers MHCII, CD40 and CD86. We detected low MHCII, CD40 and high CD86 expression in non-stimulated cells (Figure 7B, grey). Additionally, transduced cells were stimulated with LPS to gain a mature phenotype. We found a strongly increased expression of MHCII, CD40 and CD86 (Figure 7B, black) as expected.

## Discussion

Dendritic cells are one of the key players that bridge innate and adaptive immunity. Investigations concerning the properties and functions of primary dendritic cells are mainly limited due to their low number in tissue and blood. Murine bone marrow derived dendritic cells and Langerhans dendritic cells from the skin can be expanded and cultured for only a short period of time using GM-CSF [22], [23]. To overcome these limitations, we established murine inducible immortalized dendritic cells with characteristic properties of primary dendritic cells. We isolated bone marrow cells from transgenic mice with a dexamathasone and doxycyclin inducible SV40 large T-antigen and differentiated those cells into dendritic cells with GM-CSF. Dex/Dox-induced dendritic cells displayed a constant proliferation rate and can easily be expanded with a doubling time of about 70 hours. In the absence of Dex and Dox (de-induction), cells stop the large T-antigen expression and thus, lose their immortalized stage. Compared to iniDCs, de-iniDCs represent a primary dendritic cell phenotype, a slowed proliferation rate and show enhanced apoptosis and necrosis with prolonged culture (Figure 1). Several dendritic cell lines were generated during the last years. Most of them were generated by transfection or transduction with stable immortalization inducing genes. The murine bone marrow derived dendritic cell line DC2.4, retrovirally transduced with the GM-CSF transgene and the oncogenes *myc* and *raf* was generated by Shen et al. [24] and recently analyzed in more detail [25]. In addition, immortalized dendritic cells with a temperature sensitive large T-antigen had been established [26], [27]. In 2011, Baru et al. transduced murine hematopoietic stem cells with the human homeodomain transcription factor HoxB4 and differentiated those cells into functional dendritic cells [28]. Although all of those cell lines demonstrate dendritic cell properties and functionalities, they are stably immortalized. XS-52, a murine Langerhans dendritic cell line was originally isolated from the epidermis and successfully cultured in the presence of GM-CSF [29]. Although this cell line was generated without additional transgenes, it is an epidermal and mucosal-restricted dendritic cell that is not suitable for a variety of applications. Recently, Fuertes Marraco and colleagues established murine dendritic cell lines from splenic CD8α tumor cDCs, which are similar to normal splenic cDCs [30]. The advantage of our inducible immortalized dendritic cells is the complete inactivation of immortalization after de-induction, resulting in a primary-like phenotype. In the absence of Dex/Dox, the de-iniDCs express the migratory dendritic cell markers CD11c and CD11b and secrete cytokine levels equivalent to primary dendritic cells (Figure 2–3).

The iniDCs are induced with Dex/Dox, Dex being a potent immunomodulatory glucocorticoid. Several groups showed that Dex inhibits the secretion of pro-inflammatory cytokines IL-1β, IL-6, IL-12 and TNFα [31], [32]. Consequently, T cell responses induced by dendritic cells are suppressed by Dex [33], [34]. Furthermore, Dex modulates the dendritic cell maturation markers CD40, CD80, CD86 and MHCII [31], [34], [35] and CD11c (Figure 2). Indeed, we detected decreased expression of MHCII and CD11c and reduced cytokine secretion after induction of dendritic cells by Dex/Dox. However, de-induction of dendritic cells in the absence of Dex/Dox resulted in restored surface marker expression levels and cytokine secretion, comparable with BM-DCs (Figure 3–4). In contrast to our iniDCs and de-iniDCs, we could not detect an increasing MHCII expression in BM-DCs after LPS stimulation (Figure 2B). It is well known that mechanical stress during isolation and culturing of DCs results in up-regulation of MHCII [36], [37]. Importantly, the co-stimulatory molecules CD86 and CD40 were up-regulated after LPS stimulation arguing for LPS-specific maturation of BM-DCs.

Presentation of antigens to naïve T cells is an important and unique property of dendritic cells. Immature dendritic cells screen the body for pathogens and foreign molecules. Following recognition of pathogens, immature dendritic cells capture the foreign proteins, process them and present these antigens as small peptides via MHCII molecules to naïve T cells. Our 3-days cultured de-iniDCs produced high levels of pro-inflammatory cytokines. Consequently, we tested the cells for their T cell polarizing properties. In co-culture experiments with OVA-specific CD4^+^ T-cells, we demonstrated that OVA-loaded de-iniDCs induced a strong Th1, Th17 and Th2 response, detected by increased proliferation of T cells and enhanced IFNγ, IL-17 and IL-13 levels, respectively (Figure 5A–C). In addition, our de-iniDCs are able to induce CD8^+^ T cell proliferation and cytokine secretion (Figure 5D, E). Despite low level expression of CD8α on our de-iniDC, they have a strong potential of cross-presentation.

A main feature of our iniDCs is their stable proliferation under Dex/Dox treatment and their unlimited potential to switch between immortalization and the primary-like phenotype. Consequently, iniDCs are a great tool to explore detailed immunomodulatory functions or signaling pathways in dendritic cells. To elucidate novel functions, genetic engineering by retroviral gene transfer could be applied. In human plasmacytoid dendritic cells, lentiviral vectors can induce an IFNα response, which in turn activates maturation of myeloid dendritic cells [38]. Activation of lentivirally transduced myeloid dendritic cells was demonstrated by their cytokine secretion and expression of maturation markers [39]. To ensure that the immunophenotype of our dendritic cells is not altered due to infection with a viral vector, we transduced iniDCs with a lentiviral vector and investigated the characteristics of the cells. The expression of maturation markers MHCII, CD40 and CD86 of transduced iniDCs were comparable with those of non-transduced iniDCs (Figure 7B). Thus, transduced iniDCs are still inactivated after transduction with the capacity to mature with LPS stimulation, suggesting that lentiviral vector transduction does not change the phenotype of iniDCs. Alternatively, different genetic mouse models can be generated by crossing the irtTA-GBD/T-antigen mice with a mouse strain of choice, which allows e.g. the establishment of a gene specific knockout iniDC line.

Using the OTII/CD45.1 mouse model, we demonstrate that the intratracheal application of de-iniDCs elicits an innate immune response in the lungs, similar to that of primary bone marrow derived DCs. More specifically, intratracheal application of OVA loaded de-iniDCs and BM-DCs resulted in significantly higher numbers of neutrophils, detected by CD66a as a neutrophil activation marker in the BAL fluids compared to mice that received mock-treated DCs (Figure 6B) [40]. Furthermore, OVA provocation resulted in a significantly elevated number of T cells and eosinophils in the BAL fluid and infiltration of lung tissue, demonstrating the *in vivo* potential of de-iniDCs (Figure 6C–E).

In this study, we established a dendritic cell model with inducible immortalization and characteristic immune function of primary dendritic cells. After antigen uptake and presentation via MHCII, de-iniDCs activate and polarize naïve T cells into different effector cells. In the presence of LPS, iniDCs and de-iniDCs become matured, activated and produce pro-inflammatory cytokines. The presented dendritic cell line will enable functional studies to elucidate immune regulation.

## Materials and Methods

### Ethics Statement

Animal experiments were carried out in strict accordance with the German Animal Welfare Act. The protocol was approved by the Committee on the Ethics of the Landesdirektion Dresden (Permit Number: 24-9168.11-1/2010-34).

### Animals

Transgenic irtTA-GBD mice (Immorto-mouse) backcrossed to C57/BL6 background contain the SV40 large T-antigen under control of a tetracycline inducible promoter and the codon-optimized reverse tetracycline transactivator (irtTA) fused to the ligand-binding domain of a mutated glucocorticoid receptor under control of the ubiquitous expressed CAG promoter [41]. Transgenic irtTA-GBD mice, OTII/CD45.1 transgenic mice (OVA peptide 323–339-specific T cell receptor; a kind gift of Claudia Waskow (CRTD, Dresden)), and OTI transgenic mice (OVA peptide 257–264-specific T cell receptor; a kind gift of Rolf Jessberger) were bred under pathogen-free conditions.

### Preparation of bone marrow cells and generation of dendritic cells

Bone marrow cells were isolated from femur and tibia by flushing the bones with PBS containing 0.5% BSA (Sigma Aldrich) and 2 mM EDTA (Sigma Aldrich). After red blood cell lyses with ACK lysis buffer (Life Technologies), whole bone marrow cells were counted. Finally, cells were cultured in complete RPMI medium (PAA laboratories) supplemented with 10% fetal bovine serum (FBS; Thermo Scientific), 2 mM L-glutamine, 100 IU/ml penicillin, 100 µg/ml streptomycin (PAA laboratories), 1 mM sodium pyruvate, 10 mM HEPES (Biochrom AG) and 50 µM β-mercaptoethanol (Sigma Aldrich). For differentiation into dendritic cells, granulocyte macrophage colony-stimulating factor (GM-CSF; 50 ng/ml) produced by a B16 melanoma cell line [42] was added to the cell culture for 7 days. For the induction of large T-antigen expression, cells were treated with dexamethasone (Dex; 100 nM) and doxycycline (Dox; 1 µg/ml) simultaneously, leading to immortalized DCs (iniDCs). For subsequent culture, only suspension cells were transferred to new culture flasks. In further passages, GM-CSF was reduced stepwise down to 10 ng/ml. Cells were cultured at 37°C in a humidified atmosphere with 5% CO_2_.

### Generation of single cell clones

The iniDCs were counted, centrifuged and adjusted to 50 cells/20 ml medium per 96 well plate supplemented with GM-CSF (10 ng/ml), Dex (100 nM) and Dox (1 µg/ml). Single cell clones were established by limiting dilution in 96-well plates. Plates were monitored microscopically for the appearance of cell colonies and supplemented with GM-CSF, Dex and Dox. When cells reached confluency, they were trypsinized with TrypLE select (Life Technologies) for 5 minutes at 37°C. Trypsinization was terminated by dilution in medium and cells were subsequently transferred to a 48-well plate. In the following passages cells were scraped without using trypsin. When single cell clones reached 6-well formats, cells were frozen in liquid nitrogen or were directly tested for their dendritic cell properties.

### Stimulation of dendritic cells and intracellular cytokine detection

Dendritic cells were stimulated 24 hours with the TLR4 ligand lipopolysaccharide (LPS; Sigma Aldrich). For intracellular cytokine detection, the intracellular protein transport inhibitor Monensin (Biolegend) was added to the cell culture 4 hours after addition of LPS. Cells were permeabilized and fixed with Cytofix/Cytoperm solution (BD Biosciences) for 20 minutes at room temperature. Subsequently, cells were washed twice with Cytofix/Cytoperm wash buffer (BD Biosciences) and directly stained for intracellular IL-12 with the mAb IL-12p35-PE (R&D systems). Subsequently, flow-cytometric analysis was performed with an LSRII flow cytometer (BD Biosciences) and FlowJo software (Tree Star Inc.).

### Flow cytometry and cytometric bead array

Dendritic cells were stained with antibodies to analyze their surface molecule expression. The following antibodies were used: CD11c-APC, B220-PE, MHCII-FITC (Miltenyi Biotec), CD8α-APC-Cy7, CD11b-V500, Ly6C-PerCP-Cy5.5, CD86-PE-Cy7 and CD40-PE-Cy5 (BD Biosciences). For analysis of the cell composition in the bronchoalveolar lavage, the following antibodies were used: CD66a-APC (ebioscience), CD45.1-V450, Ly6C-PerCP-Cy5.5, Ly6G-FITC, CD19-PE-Cy7, CD3-V500 (BD Biosciences). Flow cytometry was performed with an LSRII flow cytometer and FlowJo was used for data analysis.

Cytokine secretion (IL-1β, IL-2, IL-4, IL-5, IL-6, IL-12p70, IL-13, IL-17, IFNγ and TNFα) was quantified with CBA Flex Sets (BD Biosciences) in the supernatant of activated cells. CBAs were measured with the LSRII and analyzed with the FCAP Array software (BD Biosciences). Cytokine levels were normalized to standard curves of recombinant cytokines.

### Antigen-presentation studies

Dendritic cells were incubated with Ovalbumin (OVA; 13.5 µg/ml; Sigma Aldrich) for 24 hours. Because the used OVA was not endotoxin-free, no additional adjuvant such as LPS was needed. Splenocytes of OTII/CD45.1 or OTI mice were isolated and CD4^+^ or CD8^+^ T cells were separated by magnetic beads (CD4 T cell isolation Kit, CD8 T cell isolation kit II, Miltenyi Biotech). OVA-loaded dendritic cells were centrifuged, washed thoroughly with PBS and co-cultured in a 1∶10 ratio (dendritic cells: T cells) in 96-well round bottom plates for 48 hours. Finally, supernatant was analyzed with CBAs for secreted cytokines.

### Intratracheal application of dendritic cells and analysis of the bronchoalveolar lavage

De-iniDCs and CD11c^+^ BM-DCs were loaded with (13.5 µg/ml) or w/o OVA for 24 hours. De-iniDCs and BM-DCs respectively were applied intratracheally to anaesthesized OTII/CD45.1 transgenic or C57BL/6 mice (1×10^6^ cells in 80 µl PBS per mouse). In detail, a 24 gauge catheter was inserted under direct vision through the vocal cords. During 3–4 spontaneous breaths, mice ‘inhaled’ the cell suspension. One group of mice received PBS without cells. After 48 hours, mice were sacrificed; 500 µl PBS was applied to the lungs via the trachea and bronchoalveolar lavage (BAL) was collected. BAL fluid was centrifuged; cells were counted and stained for flow cytometric analysis. CD66a^+^ neutrophils were analyzed by gating on CD45.1^+^, Ly6C^+^ and Ly6G^+^, and concomitant CD3 and CD19 exclusion. To induce asthma, mice received 1% w/v OVA aerosol via a nebulizer (PARI JuniorBOY®S, PARI GmbH) ten days after intratracheal cell application. For cytospin preparation, 2×10^4^ cells were stained with May-Grünwald-Giemsa. Lungs were isolated, fixed in 1% formaldehyde and embedded in paraffin. Lung sections (5 µm) were stained with Hematoxylin and Eosin (Sigma Aldrich).

### Proliferation, apoptosis and cell cycle analysis

For cell proliferation analysis, cells were stained with trypan blue (Sigma Aldrich) and counted using a haemocytometer. In parallel, cells were labeled with 1µM carboxyfluorescein diacetate succinimidyl ester CFSE (Life Technologies) and measured by flow cytometry.

To quantify apoptosis and necrosis, cells were stained with anti-Annexin V-PE antibody (BD Biosciences) and DAPI (Sigma Aldrich) for 15 min and analyzed by flow cytometry. For cell cycle analysis, cells were stained with Propidium iodide (PI; Sigma Aldrich). Briefly, cells were fixed with ice-cold ethanol (70%) for 30 minutes on ice. Subsequently, cells were washed twice with PBS, resuspended in the staining solution containing 50 µg/ml PI and 100 µg/ml RNAse A (Life Technologies) and incubated for 15 minutes at 37°C. Finally, cells were analyzed by flow cytometry.

### Transduction of dendritic cells with lentiviral vector particles

Lentiviral TRIPZ vector (Qiagen) containing turbo red fluorescence protein (turboRFP) and Puromycin resistance cassette was a kind gift of Christina Neske (University Clinic Frankfurt, pharmazentrum frankfurt, Frankfurt/Main). For generation of lentiviral vector particles, HEK293T cells were transfected with the lentiviral transfer vector, the packaging plasmid pSPAX and the envelope plasmid VSV-G in the presence of polyethylenimine (PEI, Sigma Aldrich). Virus vector particle-containing supernatant was collected after 24 hours and frozen at −80°C [43]. Dendritic cells were seeded at 2×10^5^ on RetroNectin®-coated 12-well plates and cultured for 24 hours. Then, medium was removed and replaced 1∶1 with lentiviral vector particle-containing supernatant. Subsequently, plates were centrifuged at 800 *g* for 30 minutes at 32°C and incubated for 6–8 hours at 37°C. Finally, medium was replaced by dendritic cell growth medium supplemented with Dex, Dox and GM-CSF. Transduction efficiency was determined by microscopic analysis and flow cytometry (turboRFP) after 24 hours. Transduced cells were selected in the presence of 7.5 µg/ml puromycin (InvivoGen) for 24–48 hours.
